# 

**Published:** 1884-06

**Authors:** 


					﻿Vol. IV.
JUNE, 1884.
NO. 6.
JPHE
-MEOPATHIC PHYSICIAN,
A MONTHLY JOURNAL OF MEDICAL SCIENCE.
“ Seek the Truth: come whence it may, cost what it will."
IE. CT. LIE IE, UVE. ID.,
EDITOR.
CONTENTS:
(See inside of Cover.)
PHILADELPHIA:
No. 3109 CHESTNUT STREET.
ZtSTZE'W YORK:
A. L. CHATTERTON, PUBLISHING COMPANY, PUBLISHEB’S AGENTS.
X. O 2ST 13 O TT :
ALFRED HEATH & CO., Sole Agents fob Gbeat Bbitain,
114 Ebury Street, Eaton Square.
Yearly Subscription, $2.50, invariably in advance.
Entered at the Philadelphia Post-Office as Second-Class Mail Matter.
				

